# Mapping diurnal variations in choroidal sublayer perfusion in patients with idiopathic epiretinal membrane: an optical coherence tomography angiography study

**DOI:** 10.1186/s40942-019-0162-2

**Published:** 2019-05-21

**Authors:** Felix Rommel, Fynn Siegfried, Jan A. M. Sochurek, Matthias Rothe, Max P. Brinkmann, Maximilian Kurz, Michelle Prasuhn, Salvatore Grisanti, Mahdy Ranjbar

**Affiliations:** 1 Department of Ophthalmology, Ratzeburger Allee 160, 23538 Lübeck, Germany; 2 Laboratory for Angiogenesis and Ocular Cell Transplantation, Ratzeburger Allee 160, 23538 Lübeck, Germany

**Keywords:** OCTA, Choroidal perfusion, Choriocapillaris, Sattler’s layer, Haller’s layer, Diurnal variation, Epiretinal membrane

## Abstract

**Background:**

Optical coherence tomography angiography (OCTA) is a non-invasive tool for imaging and quantifying the choroidal vasculature and perfusion state. In this index study, OCTA was used to investigate diurnal changes in choroidal sublayer perfusion in eyes with idiopathic epiretinal membrane (ERM) and to identify impacting factors.

**Methods:**

A prospective study was conducted on volunteers with symptomatic ERM, each of whom underwent repeated measurements of subfoveal choroidal thickness (SFCT) using enhanced-depth imaging optical coherence tomography and perfusion of choroidal vascular sublayers using OCTA at 7 a.m., 12 p.m., 4 p.m., and 8 p.m. Possible interactions between diurnal variations and other factors, such as gender and age, were evaluated.

**Results:**

A total of 21 eyes of 21 participants (mean age 72.43 ± 7.06 years) were analysed. A significant pattern of diurnal variation was observed for SFCT (*p* = 0.008) as well as perfusion of Haller’s layer (HLP, *p* = 0.001). SFCT and HLP both demonstrated a quadratic relation to time of the day, decreasing from morning to afternoon, before increasing again in the evening. No significant differences with regard to gender or age were detectable.

**Conclusion:**

OCTA-based analysis of choroidal sublayer perfusion demonstrated significant diurnal variations in patients with symptomatic ERM, which are quite different from changes reported in healthy eyes. Therefore, it is important to account for time of day, when comparing longitudinal OCTA data.

## Introduction

The choroid plays an important role in the pathogenesis of various diseases in the eye. Changes in choroidal thickness and its amount of vascularization have been found in diseases like age-related macular degeneration, central serous chorioretinopathy or diabetic retinopathy [1, 2]. With the recent development of OCTA the vascular network of the choroid can be assessed in vivo and in real time by creating slab-segmented angiograms [3]. Several OCTA-based studies already reported differences in choriocapillaris perfusion (CCP) in ocular diseases [4–7].

Diurnal variations have previously been shown for intraocular pressure (IOP), axial length (AL) and subfoveal choroidal thickness (SFCT). These studies reported a significantly thicker SFCT in the morning than in the evening [8, 9]. In choroidal substructure analyzation, Gabriel et al. [10] demonstrated that Sattler’s layer (SL), but not Haller’s layer (HL) thickness is subject to diurnal changes. Recently the first OCTA-based study showed diurnal variation of CCP similar to subfoveal choroidal thickness, being higher in the morning and lower in the evening [11].

ERM is a common disease in elderly people leading to structural changes of the macular architecture causing variable loss of visual acuity and metamorphopsia [12]. The ERM forms along the surface of the internal limiting membrane (ILM), causing vertical traction with thickening of the macula, as well as tangential forces dragging the retina from its original position and displacing the vessels [13, 14]. Yu et al. [15] demonstrated reduced CCP in patients suffering from ERM using OCTA. Furthermore several OCT- and OCTA-based studies revealed significant changes in choroidal thickness as well as perfusion following vitrectomy with ERM-ILM peeling [7, 15–17].

While SFCT in general, and CCP have become important focus in research, little is known about characteristics of SL perfusion (SLP) and HL perfusion (HLP). In this study we aimed to evaluate diurnal variations of SFCT and choroidal sublayer perfusion in patients with ERM.

## Methods

Participants for this prospective observational study were recruited from the Department of Ophthalmology at the University of Lübeck. The study was approved by the institutional review board and was conducted in accordance with the Declaration of Helsinki. All subjects received detailed information about the study and written informed consent was obtained individually by each participant before enrolment. Only eyes with idiopathic, symptomatic ERM affecting the fovea as validated via SD-OCT and presence of metamorphopsia, which were scheduled for surgery on the next day were included. The contralateral eye was unaffected or did also present with ERM or was subject to ERM surgery in the past and did not undergo further investigation. Any other history of ocular or cardiovascular disease, antihypertensive drug use, as well as diabetes mellitus was defined as exclusion criteria. Ethnically all participants were Caucasian and they underwent a thorough examination including blood pressure (BP), refraction, best-corrected visual acuity (BCVA) in Snellen, IOP, AL, slit-lamp biomicroscopy, macular EDI-OCT as well as OCTA. The maximum permissible spherical and cylindrical aberration was ± 3 and ± 1 diopters respectively.

Imaging was performed on all subjects without prior pupil dilatation using the HS-100 (Canon, Tokyo, Japan) OCT/OCTA device at 7 a.m., 12 p.m., 4 p.m. and 8 p.m. by a single, trained operator. Each imaging session included EDI-OCT scans (10 × 10 mm^2^) and OCTA (3 ×  3 mm^2^) volumetric scans of the posterior pole. The HS-100 device works with a modified full-spectrum amplitude decorrelation algorithm to generate flow maps. Only images of high quality without motion artifacts were accepted to guarantee standardized analysis.

SFCT was measured manually in EDI-OCT scans just below the fovea, extending perpendicularly from the hyperreflective Bruch’s membrane layer to the inner scleral border. Manual measurements were performed by at least two experienced graders (FR, MRo and MPB) who were blinded to the clinical information of the examined eyes.

**Fig. 1:**
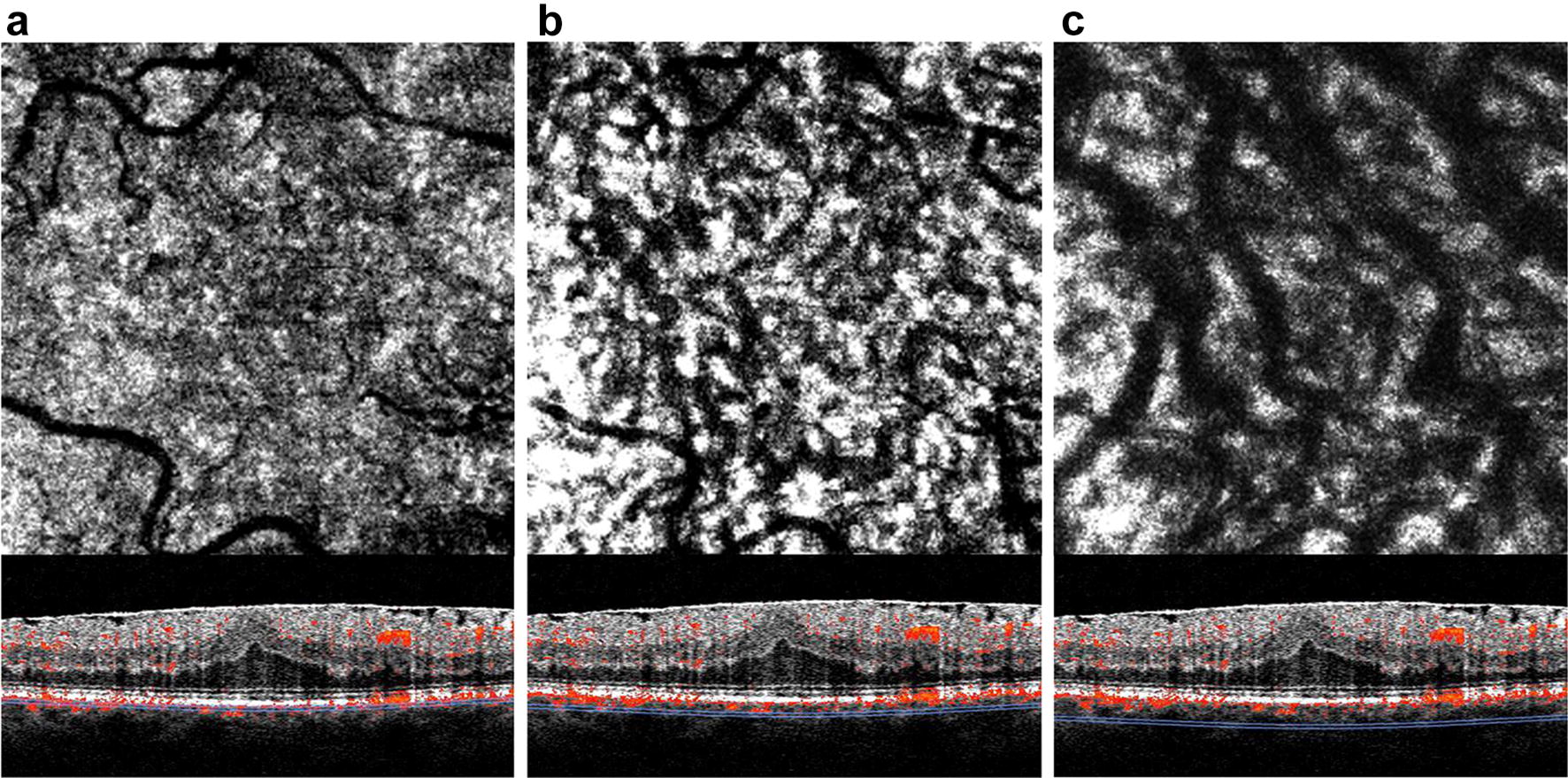
OCTA-Imaging of the posterior pole with ERM. Angiogram and corresponding B-scan at the level of the choriocapillaris (**a**), Sattler’s layer (**b**), and Haller’s layer (**c**)

After acquisition, OCTA data were manually segmented in all B-scans to get 20 µm slabs of CC (Fig. 1a), SL (Fig. 1b) and HL (Fig. 1c) according to previous published protocols [10, 18, 19]. Each en face image was exported into ImageJ (NIH, Version 1.48b, Bethesda, USA) and binarized by the Otsu method, which is an automatic threshold selection from grey-level histograms, to determine the percentage of white and black pixels [20]. As suggested by Nicolò et al., CCP was calculated by scoring the percentage of white pixels, while for SLP and HLP the percentage of black pixels was taken into account [19].

Statistical analyses were performed using IBM SPSS (Version 24.0, Chicago, IL, USA) and Prism GraphPad (Version 8.0, La Jolla, CA, USA). BCVA measurements in decimal Snellen were converted to logarithm of the minimum angle of resolution (logMAR). Mean arterial pressure (MAP) was calculated based on systolic and diastolic BP (2/3 diastolic BP + 1/3 systolic BP). The Shapiro–Wilk test was used to check for normality of all obtained data. Diurnal changes in MAP, IOP, SFCT, CCP, SLP and HLP were evaluated using a repeated measures multivariate analysis of variance (MANOVA). A two-way mixed-model MANOVA was performed to investigate age- as well as gender-dependent interactions. For all tests values of *p* < 0.05 were considered statistically significant.

## Results

A total of 21 eyes of 21 patients with ERM were included in the analysis. Demographic and clinical data are reported in Table 1.

**Table 1:** Demographic and clinical data

Parameter	Mean ± SD	Median (min; max)
Age (years)	72.43 ± 7.06	74 (56; 86)
Sex (F/M)	13 (61.9%)/8 (38.1%)	
Axial length (mm)	23.75 ± 1.01	23.75 (21.9; 25.54)
BCVA (logMAR)	0.33 ± 0.21	0.3 (0.1; 1.0)

*F* female, *M* male, *SD* standard deviation, *BCVA* best-corrected visual acuity

**Fig. 2:**
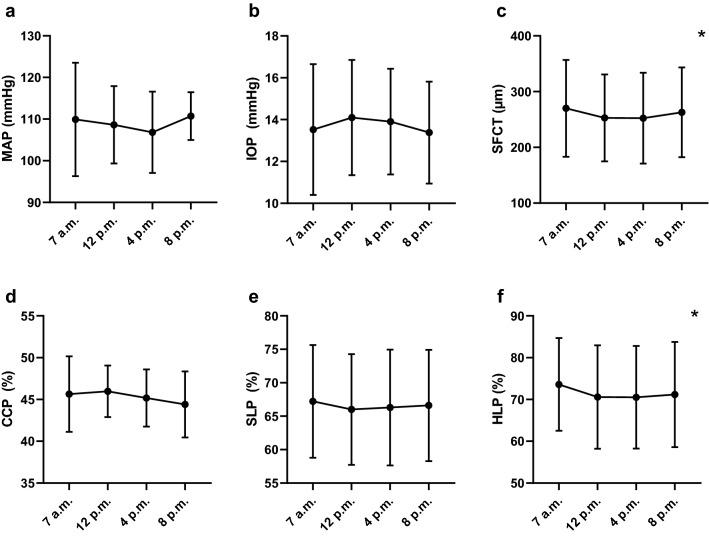
Diurnal changes in **a** mean arterial pressure (MAP), **b** intraocular pressure (IOP), **c** subfoveal choroidal thickness (SFCT), **d** choriocapillaris perfusion (CCP), **e** Sattler’s layer perfusion (SLP) and **f** Haller’s layer perfusion (HLP) are illustrated. Values are expressed as the mean with standard deviation at 4 time points during the day. Variables showing statistically significant diurnal changes are indicated with an asterix

Overall analysis showed statistically significant diurnal changes [F(18, 156.05) = 2.56, *p* = 0.001, partial η^2^ = 0.216]. Univariate analysis with Greenhouse–Geisser correction indicated significant diurnal variations for SFCT [F(2.45, 48.91) = 4.79, *p* = 0.008, partial η^2^ = 0.193] and HLP [F(1.98, 39.64) = 8.33, *p* = 0.001, partial η^2^ = 0.294]. SFCT was found to be the thickest in the morning (269.76 ± 86.73 µm), declining until afternoon (252.17 ± 81.57 µm) before increasing slightly in the evening again (262.76 ± 80.45 µm) (Fig. 2c). HLP showed similar diurnal variation with the highest perfusion state in the morning (73.6 ± 11.07%), decreasing until afternoon (70.53 ± 12.29%) and increasing marginally in the evening (71.17 ± 12.59%) (Fig. 2f). MAP [F(2.49, 49.80) = 0.94, *p* = 0.416, partial η^2^ = 0.045], IOP [F(2.60, 52) = 0.64, *p* = 0.574, partial η^2^ = 0.031] as well as CCP [F(2.38, 47.49) = 2.29, *p* = 0.104, partial η^2^ = 0.103] and SLP [F(2.46, 49.21) = 1.37, *p* = 0.264, partial η^2^ = 0.064] did not show significant diurnal fluctuations (Fig. 1a, b, d, e).

Mixed-model MANOVA did not reveal any significant interaction between gender and time of day as both genders showed significant diurnal changes in choroidal characteristics [F(18, 147.56) = 2.58, *p* = 0.001, partial η^2^ = 0.227]. Univariate analyses with Greenhouse–Geisser correction demonstrated significant diurnal fluctuations for SFCT [F(2.47, 46.89) = 3.85, *p* = 0.021, partial η^2^ = 0.169] and HLP [F(2.03, 38.62) = 9.22, *p* = 0.001, partial η^2^ = 0.327] for both genders, but not for CCP [F(2.32, 44.11) = 2.26, *p* = 0.109, partial η^2^ = 0.106] and SLP [F(2.39, 45.38) = 1.71, *p* = 0.188, partial η^2^ = 0.082].

Further analysis did not reveal any significant interaction between age (≤ 75 years vs. > 75 years) and time of the day as both age groups showed significant diurnal changes [F(18, 147.56) = 3.11, *p* < 0.001, partial η^2^ = 0.262]. Univariate analyses with Greenhouse–Geisser correction demonstrated significant diurnal fluctuations for SFCT [F(2.58, 47.11) = 4.79, *p* = 0.008, partial η^2^ = 0.201] and HLP [F(2.02, 38.34) = 8.79, *p* = 0.001, partial η^2^ = 0.316] for both age groups, but not for CCP [F(2.52, 47.95) = 2.62, *p* = 0.071, partial η^2^ = 0.121] and SLP [F(2.44, 46.34) = 1.13, *p* = 0.341, partial η^2^ = 0.056].

## Discussion

This OCTA-based study demonstrated significant diurnal variations in SFCT and HLP in subjects with ERM. SFCT and HLP both showed a quadratic relation to time of the day, decreasing from morning to afternoon, before slightly increasing again in the evening. Previous investigations have shown diurnal variations in SFCT and choroidal perfusion in subjects without any ocular disease, but to our knowledge this is the first study demonstrating diurnal changes in choroidal metrics in eyes with ERM [8, 9, 21].

In healthy subjects a progressive decrease in SFCT during the day is reported. Tan et al. found significant circadian changes in SFCT with the highest thickness at 9 a.m., linear decreasing to the latest time point (5 p.m.) [9]. A similar pattern was observed by Usui et al. demonstrating a thinning of the SFCT during the day and a thickening in the night [21]. Our study also demonstrated significant diurnal changes of SFCT in subjects with ERM with thinning during the day, but thickness increase from afternoon to evening.

The influence of ERM on choroidal thickness has been described by Michalewska et al., who demonstrated a decrease of SFCT 3 month after vitrectomy with ERM removal and ILM peeling [16]. They even hypothesized that generalized vascular changes and increased SFCT may contribute to the formation of ERMs. However the present study emphasized the influence of ERM on SFCT by changes on the usual diurnal pattern of SFCT. It seems likely that the anteroposterior and tangential forces due to ERM create retinal traction and distortion with choroidal involvement as well.

Recently Sarwar et al. [11] published the first study evaluating diurnal changes in CCP by using OCTA. They reported a significant decrease of vessel flow density in CC from 9 a.m. to 6 p.m. The change in CCP also correlated positively with change in SFCT. However, the authors drew their conclusion based on the evaluation of only two time points during the day. Thereby they might have missed out on other changes of CC blood flow during the day. Furthermore they did not evaluate SLP and HLP, as, to the best of our knowledge, so far diurnal changes in choroidal sublayer perfusion have not been evaluated using OCTA. Nevertheless we did not observe diurnal variation in CCP over four time points. From this we conclude that ERM seems to have significant influence on the blood flow at least in CC. Yu et al. support these findings as they reported a reduced CCP in ERM eyes compared to the unaffected fellow eyes, which was reversible by surgery [15]. Likewise, Chen et al. [7] reported reduced CCP in ERM eyes, but without a significant increase after surgery. The authors assumed different vessel distortion in retina and choroid by ERM-associated traction. Vessels in the superficial and deep capillary plexus seem to expand due to the centripetal force of ERM, which may subsequently influence the microvasculature in CC. This would be a possible explanation for the suspended diurnal variation of CCP in ERM eyes. It remains unclear to what extent ERM-associated traction influences the microvasculature in SL and HL, since the vessel size increases from CC to HL [18]. To the best of our knowledge this is the first study reporting significant diurnal changes of HLP in subjects with ERM, but not for CCP and SLP.

Since choroidal vessels seem to have some autoregulatory capacity, the influence of MAP must be taken into account [21]. While Straubhaar et al. did not report significant influence of MAP on the choroidal laser doppler flowmetry parameters, several reports demonstrated the dependence of choroidal blood flow on MAP and even IOP [22–24]. However, in the current study we did not observe significant diurnal changes neither in MAP, nor in IOP.

Previous studies also demonstrated gender-dependent differences in SFCT. This could be explained by the finding of androgen as well as estrogen receptors in the choroid [25, 26]. In our study we did not find any significant differences in choroidal metrics between female and male, as both genders equally showed significant diurnal changes in SFCT and HLP. This could be due to our older subjects (mean age: 72.43), as the hormonal status, especially with regards to sex hormones, changes during aging [27].

Capillaries are subject to age-related morphological and functional changes, such as decreasing vessel density and diameter as well as vascular stiffening and restricted autoregulation [28, 29]. All of these findings may lead to fewer diurnal fluctuation of choroidal blood flow with increasing age. However, in our study we couldn’t demonstrate any significant differences in choroidal metrics between subjects ≤ 75 years and subjects above, as they both showed significant diurnal variation in SFCT and HLP. From this one could draw conclusion that ERM could possibly influence the natural aging process of the choroid. Though, because of the narrow range of age and the nature of this elderly population, differences between age groups were not to be expected.

The present study has some limitations. As we only examined participants during daytime from 7 a.m. to 8 p.m., we may have missed out on important information of the choroidal perfusion during the night. Furthermore, the manual measurement of SFCT may represent a potential bias. To reduce this possible confounding factor, all measurements were performed in a masked fashion by two experienced graders and the average values were used for statistical analysis. In addition, subjects with ERM are prone to segmentation errors in OCTA, as ERM is erroneously interpreted as ILM, which are typically more prevalent in cases with a distinct and hyper reflective ERM [30]. Prior to quantitative analysis of retinal vessels in subjects with ERM, the corresponding B-scans should be checked for plausibility and segmentation errors must be manually corrected to avoid incorrect measurements. However, the high contrast of the retinal pigment epithelium (RPE) allows a robust segmentation, thus segmentation errors don’t play a significant role in analyses of the choroid. By using a single OCTA device our methodical approach is restricted as perfusion values differ from device to device, depending on hardware, segmentation, as well as software algorithm and it remains to be tested whether our finding of diurnal changes in choroidal sublayer perfusion in patients with ERM can be validated using various commercially available OCTA devices. In addition it should be noted that we only used the Otsu image processing algorithm and another algorithm might have led to a divergent outcome as it has been shown that algorithms do have different discriminatory abilities [31]. Finally, the sample size may represent a potential limiting factor leading to a mainly exploratory data analysis. Nevertheless, strong significant diurnal variations were found. To corroborate our findings, further studies with a larger number of participants will be necessary.

## Conclusion

In conclusion, OCTA is becoming an important non-invasive tool for imaging and quantifying the choroidal vasculature and perfusion state. It is important to account for time of the day, when comparing longitudinal OCTA data of patients with ERM. The present study indicates an influence of ERM on the choroid in terms of changes in the usual diurnal pattern of SFCT and choroidal perfusion. Further studies are needed to assess, whether this finding might prove any significance in the pathophysiology of ERMs.

## Abbreviations

AL: axial length
BCVA: best-corrected visual acuity
BP: blood pressure
CC: choriocapillaris
CCP: choriocapillaris perfusion
EDI-OCT: enhanced-depth imaging optical coherence tomography
ERM: epiretinal membrane
HL: Haller’s layer
HLP: Haller’s layer perfusion
ILM: internal limiting membrane
IOP: intraocular pressure
MANOVA: multivariate analysis of variance
MAP: mean arterial pressure
OCTA: optical coherence tomography angiography
SFCT: subfoveal choroidal thickness
SL: Sattler’s layer
SLP: Sattler’s layer perfusion
